# Experimental Study on Activated Carbon-MIL-101(Cr) Composites for Ethanol Vapor Adsorption

**DOI:** 10.3390/ma14143811

**Published:** 2021-07-08

**Authors:** Zhongbao Liu, Jiayang Gao, Xin Qi, Zhi Zhao, Han Sun

**Affiliations:** 1 Department of Refrigeration and Cryogenic Engineering, College of Environmental and Energy Engineering, Beijing University of Technology, 100 Pingleyuan Road, Chaoyang, Beijing 100124, China; gaojiayang@emails.bjut.edu.cn (J.G.); zhaoz@emails.bjut.edu.cn (Z.Z.); h.sun@bjut.edu.cn (H.S.); 2 China Household Electric Appliance Research Institute, 6 Yuetan Beixiao Str., Xicheng, Beijing 100037, China; qix@cheari.com

**Keywords:** MIL-101(Cr), activated carbon, adsorption, ethanol vapor, kinetic analysis

## Abstract

In this study, the hydrothermal method was used to synthesize MIL-101(Cr), and activated carbon (AC) with different content was incorporated in to MIL-101(Cr), thereby obtaining AC-MIL-101(Cr) composite material with a huge specific surface area. The physical properties of MIL-101(Cr) and AC-MIL-101(Cr) were characterized by powder X-ray diffraction (PXRD), scanning electron microscopy (SEM), thermogravimetric analysis (TGA), nitrogen adsorption and desorption and specific surface area testing, and ethanol vapor adsorption performance testing. The results show that with the increase of activated carbon content, the thermal stability of AC-MIL-101(Cr) is improved. Compared with the pure sample, the BET specific surface area and pore volume of AC-MIL-101(Cr) have increased. In the relative pressure range of 0–0.4, the saturated adsorption capacity of AC-MIL-101(Cr) to ethanol vapor decreases slightly. It is lower than MIL-101(Cr), but its adsorption rate is improved. Therefore, AC-MIL-101(Cr)/ethanol vapor has a good application prospect in adsorption refrigeration systems. The exploration of AC-MIL-101(Cr) composite materials in this paper provides a reference for the future application of carbon-based/MOFS composite adsorbent/ethanol vapor working fluid in adsorption refrigeration.

## 1. Introduction

Electricity consumption caused by the demand for cooling and heating, as a result of the continuous development of the social economy, is expected to increase tenfold from 2010 to 2100 [1]. In response to the global call for energy conservation and emission reduction, it is particularly important to explore and develop energy-saving technologies that utilize low-grade thermal energy, such as solar energy and industrial waste heat. Traditional compression cooling systems consume a large amount of high-grade driving energy while cooling and heating. Adsorption heat pumps (AHPs) use adsorption beds instead of compressors to achieve cooling and heating purposes during the process of adsorbent adsorption and desorption and adsorbate evaporation and condensation. Overcoming the limitations of mechanical compression systems and liquid–vapor absorption systems, it has the advantages of being able to use low-grade heat, no moving parts, and solid adsorbents with good heat storage performance. Most importantly, it can be adapted to the needs of different applications by adjusting the combination of adsorbent/refrigerant working fluid pairs [2]. At present, the more traditional working fluid pairs include activated carbon/ammonia, activated carbon/methanol vapor, zeolite/water, silica gel/water, etc., which usually exhibit medium adsorption capacity and/or extremely high adsorption enthalpy, which cannot meet the requirements of high-performance AHPs [3]. Therefore, exploring working fluids with stable performance and good absorption and desorption performance has become an important direction for the study of adsorption heat pumps. Among the common refrigerants, ethanol vapor has the characteristics of low freezing points (−114 °C) that water does not have, which satisfies its application under low temperature conditions; compared with methanol vapor, ethanol vapor is non-toxic, ecologically compatible, and compatible with various materials, but its latent heat is 30% lower than methanol vapor.

Among the common adsorbents, activated carbon has many sources and is low in price, and activated carbon has a large adsorption capacity for refrigerant ethanol vapor, and its desorption temperature and adsorption heat are low. Therefore, there are a large number of related reports on the application of activated carbon-ethanol vapor to adsorption refrigeration. A. Frazzica and others used the activated carbon/ethanol vapor working pair to evaluate the thermodynamic performance that can be achieved under air conditioning and refrigeration conditions through the design, implementation, and testing of a new small adsorption refrigerator prototype, and carried out experimental activities to determine the market The best activated carbon available [4]. I.I. El-Sharkawy et al. [5] compared the adsorption performance of high-porous activated carbon Maxsorb III-ethanol vapor and activated carbon fiber (ACF)-ethanol vapor working fluid pairs. Experimental results show that Maxsorb III can adsorb 1.2 kg of ethanol vapor per kg of adsorbent. Theoretical calculations show that the specific cooling power (SCP) of Maxsorb III-ethanol vapor working fluid pair is 420 kJ/kg when the evaporation temperature is 280 K and the desorption temperature is 353 K.

In recent years, metal-organic frameworks (MOFs) have been rapidly developed as a new type of adsorbent with excellent performance. This new type of porous material is freely assembled by metal clusters and organic ligands, with ultra-high specific surface area (up to 6000 m^2^/g [6]) and porosity. Moreover, its pore structure is adjustable. These excellent properties mean MOFs have broad potential application, such as heterogeneous catalysis [7], drug delivery [8], sensor technology [9], and especially gas adsorption and storage.

Rezk [10] and Janiak [11] studied the ethanol vapor adsorption performance of MIL-101(Cr) and MIL-100 (Cr), and the results showed that the ethanol vapor adsorption isotherms of MIL-101(Cr) could be regarded as two combinations of I-type isotherms, one of which occurs in the low-pressure stage and the other in the medium pressure stage. In addition, after 20 adsorption–desorption cycles, the structure can still remain intact. Although the adsorption interval of MIL-100 (Cr) occurs at a lower pressure, the saturated adsorption capacity is much lower than that of MIL-101(Cr). When the adsorption temperature is 303 K, 1 kg MIL-101(Cr) can adsorb 1.1 kg ethanol vapor.

Ma et al. [12] studied the adsorption refrigeration performance and cyclic adsorption stability of the molded MIL-101-ethanol vapor on an adsorption refrigeration simulation device. The results show that: at 298 K, the equilibrium adsorption capacity of ethanol vapor on the molded MIL-101(Cr) is 0.74 kg/kg, the desorption peak temperature is 327 K, and the complete desorption temperature is about 373 K. When the desorption temperature is 353 K, MIL-101(Cr)-ethanol vapor working pair has a cooling capacity of 283 kJ/kg, which is 2.2 times that of activated carbon–ethanol vapor. After 60 cycles, the formed MIL-101(Cr) has no significant decrease in ethanol vapor absorption, and its Brunauer–Emmett–Teller (BET) surface area only decreases by 3.3%.

de Lange et al. [13] investigated the applicability of 18 different MOF structures for application in adsorption-driven heat pumps (AHP) and chillers (AC) using either methanol or ethanol as the working fluid, based on adsorption measurements and thermodynamic assessment. Although the adsorption capacity of MIL-101 for methanol and ethanol is relatively high, the resolution temperature of mil is relatively high at low relative pressure, which is not suitable for use in AHPs/ACs and the most suitable MOFs identified in this work are UiO-67, CAU-3, and ZIF-8, from a thermodynamic perspective, for both methanol and ethanol.

Although both MIL-101(Cr) and activated carbon (AC) have good adsorption properties for ethanol vapor, there is currently no research on making them into composite adsorption materials and applying them to ethanol vapor adsorption. In this paper, activated carbon-MIL-101(Cr) (AC-MIL-101(Cr)) composite materials with different AC ratios (5 and 10 wt%) were prepared by an in-situ composite method and named 5% AC-MIL-101(Cr) and 10% AC-MIL-101(Cr). Through XRD, SEM, TGA, N_2_ adsorption–desorption isotherm and other characterization methods, the characteristics of AC-MIL-101(Cr), such as image, crystallinity, thermal stability, and pore structure, were studied. The adsorption performance of AC-MIL-101(Cr) on ethanol vapor was explored for the first time, indicating the application potential of AC-MIL-101(Cr)/ethanol vapor working fluid in solid adsorption refrigeration systems.

## 2. Experimental

### 2.1. Reagents and Materials

The reagents used in the experiment are as follows: Chromium (III) nitrate nine hydrate (Cr(NO_3_)_3_·9H_2_O, ≥99% purity; Braunwell Technology Co., Ltd., Beijing, China), and 1,4-benzenedicarboxylic acid (H_2_BDC, ≥99.0% purity); Beijing Sinopharm Chemical Reagent, Beijing, China), N,N-Dimethylformamide (DMF, ≥99% purity; Braunwell Technology Co., Ltd., Beijing, China), Hydrofluoric acid (HF, ≥40% purity; Tianjin Kermel Chemical Reagent Co., Ltd., Beijing, China), ammonium fluoride (NH_4_F, ≥96% purity; Beijing Sinopharm Chemical Reagent, Beijing, China), absolute ethanol vapor (≥99.7% purity; Beijing Sinopharm Chemical Reagent, Beijing, China), activated carbon (S**_BET_** = 560 m^2^/g), deionized water (homemade in the laboratory of Beijing University of Technology, Beijing, China).

### 2.2. Synthesis of MIL-101(Cr)

According to the literature [14], MIL-101(Cr) was synthesized by the hydrothermal method. The specific synthesis process went as follows: first, we put 12 g of Chromium (III) nitrate nine hydrate, 4.92 g of 1,4-benzenedicarboxylic acid, and 144 mL of distilled water in a three-necked flask, and heated and stirred the three-necked flask in a 313 K oil bath for 30 min to make it uniformly mixed. The mixture was then transferred to a Teflon lined autoclave; 1.5 mL of HF was added to the mixture. Then the mixture was heated in an oil bath at 493 K for 8 h. After the reaction was over, we took out the reactor and cooled it to room temperature. We heated and stirred the crude product (without the incompletely reacted terephthalic acid crystals) and 60 mL of N,N-dimethylformamide at 313 K for 1 h. Finally, the obtained mixture was filtered; the filtrate was centrifuged, and washed with ammonium fluoride, water, and ethanol vapor in sequence. The product obtained by centrifugation was vacuum dried at 423 K for 12 h.

### 2.3. Synthesis of AC-MIL-101(Cr)

The preparation method of AC-MIL-101(Cr) [15] went as follows: first of all, 150 mg AC (about 5 wt.% of final product of pristine MIL-101(Cr)) and 300 mg AC (about 10 wt.% of the final product of pristine MIL-101(Cr)) were attended to the solution composed of Chromium (III) nitrate nine hydrate (Cr(NO_3_)_3_·9H_2_O, 12 g), 1,4-benzenedicarboxylic acid (H_2_BDC, 4.92 g), and distilled water (144 mL), respectively. The next steps are shown in Section 2.2. By adding different contents of AC, two composite materials were obtained: 5% AC-MIL-101(Cr) and 10% AC-MIL-101(Cr).

### 2.4. Characterization

In this study, the Bruker D8 Advance X-ray diffractometer was used to characterize the crystallinity of the synthetic material. The test conditions were: at room temperature, using CuKα (λ = 0.15432 nm) rays, the scanning speed was 6°/min, and the scanning range was 5–30°. A Hitachi S-488 scanning electron microscope was used to conduct powder tests on the materials to observe the morphological characteristics of the composite materials. The thermo gravimetric analysis (Seiko TG/DTA6300) was applied to determine the thermal stability of synthetic materials. The sample dosage was about 4 mg, under argon gas flow of 20 mL/min, the heating rate was 10 K/min, and the test temperature range was: 303 K–1073 K. The N_2_ adsorption–desorption isotherm, the BET and Langmuir equations were used to calculate the BET and Langmuir specific surface area of the sample, and the BJH and HK equations were used to calculate the pore size distribution of the porous material. The test conditions were: after the sample was vacuum pretreated at 373 K for 6 h, the N_2_ absorption and desorption test was performed at 77 K (Micromeritics ASAP 2020HD88).

In this study, the adsorption isotherms and adsorption kinetics of MIL-101(Cr) and AC-MIL-101(Cr) samples were measured by 3H-2000PW gravimetric adsorption analyzer (Beishide Instrument Technology (Beijing) Co., Ltd., Beijing, China), and the adsorption effect of the samples on ethanol vapor was analyzed. Ethanol vapor adsorption measurement process: (1) pretreatment: in order to remove the water vapor in the air adsorbed by the sample and the solvent molecules in the sample crystals, the samples (approximately 40 mg) were dried at 373 K in a vacuum for 12 h. (2) We set the adsorption temperature to 303 K and the relative pressure range was 0–1.

## 3. Results and Discussion

### 3.1. Sample Yield

**Figure 1:**
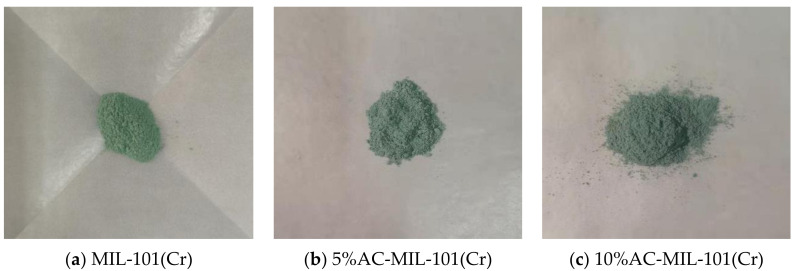
Three sample finished products.

Through the methods shown in Section 2.2 and Section 2.3, three sample finished products of MIL-101(Cr) 3.802 g, 5%-AC-MIL-101(Cr) 3.960 g, and 10%AC-MIL-101(Cr) 4.554 g were prepared. As shown in Figure 1, there is no difference in appearance of the three samples, all of which were green powder. It shows that the addition of AC has little effect on the output of the material.

### 3.2. XRD Analysis

**Figure 2:**
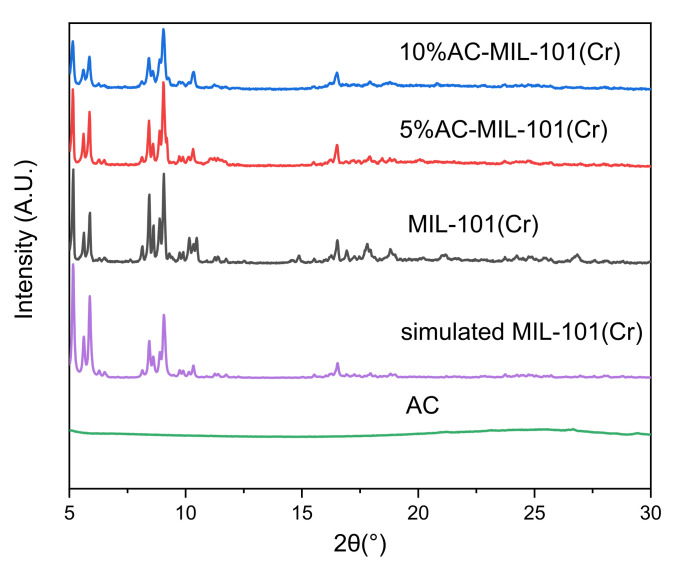
XRD pattern.

The XRD spectra of MIL-101(Cr), 5% AC-MIL-101(Cr), and 10% AC-MIL-101(Cr) are shown in Figure 2. From the XRD spectrum data, it can be clearly seen that the characteristic diffraction peak data (2θ = 5.98°, 8.56°, 9.18°, 10.30°, 16.54°) of MIL-101(Cr) fits well with the data reported in the early literature [16,17], indicating the success of MIL-101(Cr) preparation. Compared with MIL-101(Cr), the characteristic diffraction peaks of 5% AC-MIL-101(Cr) and 10% AC-MIL-101(Cr) have no change in the position of the characteristic diffraction peaks, indicating that the addition of AC will not affect the skeletal crystal structure of MIL-101(Cr), but the intensity of the characteristic diffraction peak of the composite material decreases slightly.

### 3.3. SEM Analysis

**Figure 3:**
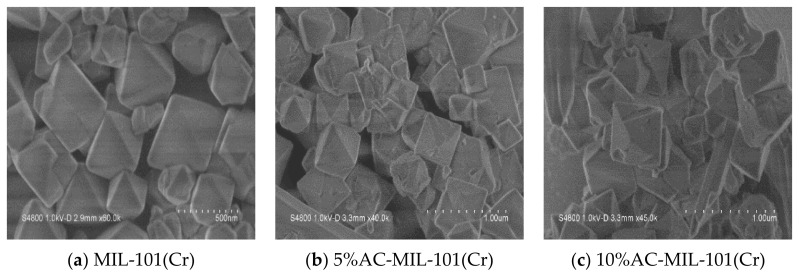
SEM images of (**a**) MIL-101(Cr); (**b**) 5% AC-MIL-101(Cr) and (**c**) 10% AC-MIL-101(Cr) samples.

The SEM image of the synthesized sample is shown in Figure 3. The MIL-101(Cr) crystal clearly presents a regular octahedral structure; 5% AC-MIL-101(Cr) crystal and 10% AC-MIL-101(Cr) crystal have the same structure as MIL-101(Cr) crystal, similar to the octahedral facade structure, but the composite crystal surface presents certain defects [18].

### 3.4. Thermogravimetric Analysis

**Figure 4:**
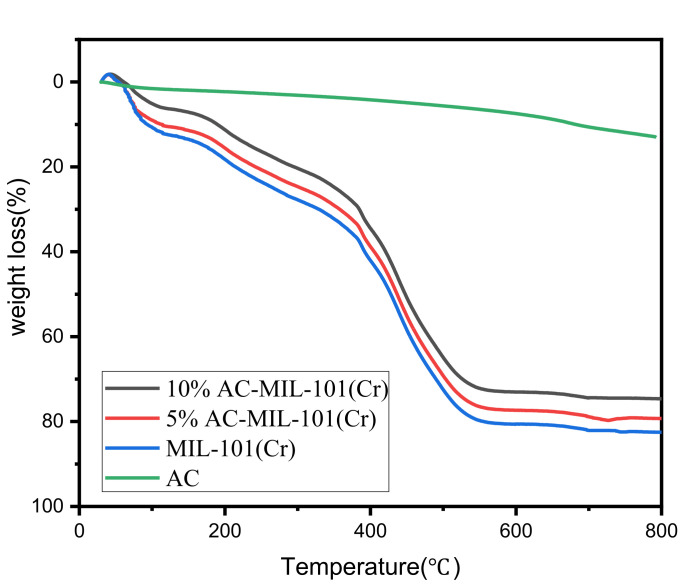
TG curves of the MIL-101(Cr) and the AC-MIL-101(Cr) composites.

The thermal stability of the material was studied by thermogravimetric analysis (TGA). Figure 4 shows the thermogravimetric analysis results of the three materials. The test results fit the data in the literature [15]. Each material exhibits three steps: the weight loss in the first two stages may be due to adsorption. The water molecules in MIL-101(Cr) and AC-MIL-101(Cr) desorbed from the large and small cages of MIL-101(Cr) and AC-MIL-101(Cr) when heated, and the weight loss at this time was about 30%. The first stage was from 303 to 393 K, the weight loss was about 13%; the second stage was from 423 to 623 K, the weight loss was about 17%. For the last stage, from 643 to 823 K, the weight loss was about 50%, which was caused by the heat elimination of OH/F groups in the MIL-101(Cr) and AC-MIL-101(Cr) frameworks [19]. Compared with MIL-101(Cr), AC-MIL-101(Cr) has less weight loss and better thermal stability at the same temperature.

### 3.5. Surface and Pore Analysis

**Figure 5:**
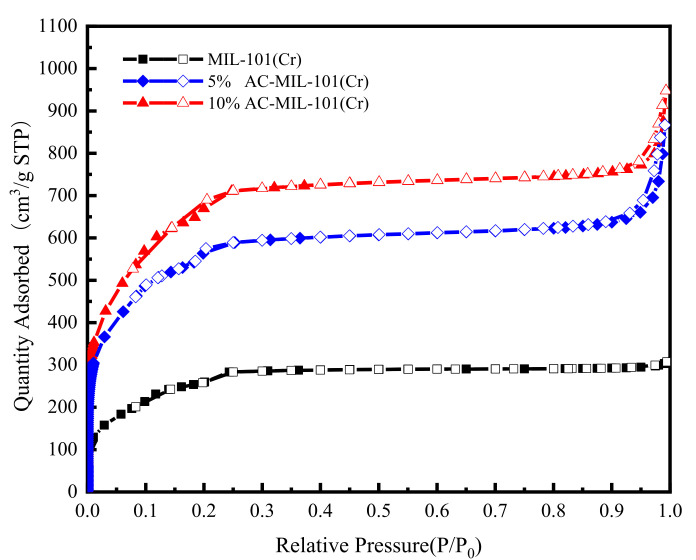
N_2_ adsorption–desorption isotherms measured for MIL-101(Cr), 5% AC-MIL101(Cr) and 10% AC-MIL-101(Cr). (Filled symbols represent adsorption and open symbols represent desorption).

**Figure 6:**
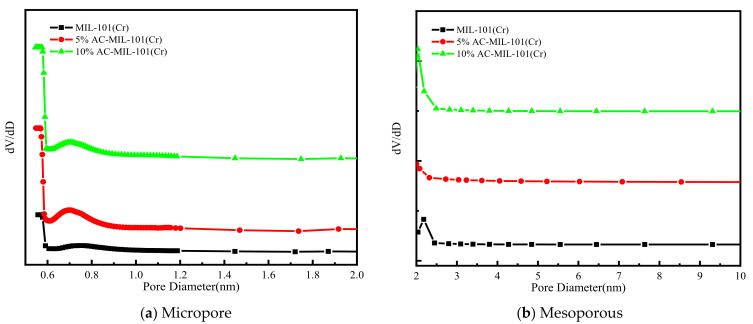
Pore diameter distribution of MIL-101(Cr) and AC-MIL-101(Cr): (**a**) micropore; (**b**) mesoporous.

Figure 5 shows the N_2_ adsorption–desorption isotherms of MIL-101(Cr) and AC-MIL-101(Cr) materials at 77 K. At 77K, the N_2_ adsorption–desorption isotherm of the composite material AC-MIL-101(Cr) has basically the same shape as the original MIL-101(Cr) N_2_ adsorption–desorption isotherm, showing an I-type isotherm. Under low relative pressure, the adsorption capacity of the three samples on N_2_ increased sharply. AC-MIL-101(Cr) has a second adsorption at a relative pressure of 0.15–0.2, while MIL-101(Cr) has a second adsorption at a relative pressure of 0.2–0.25, indicating that the synthesized samples contain two kind of different cages [20]. Compared with the original MIL-101(Cr), the equilibrium adsorption capacity of AC-MIL-101(Cr) has been greatly improved, and with the increase of the activated carbon content, the greater the improvement effect of the composite material on the adsorption performance of N_2_. The equilibrium adsorption capacity of MIL-101(Cr) is about 260 cm^3^/g, and the maximum adsorption capacity is 307.3 cm^3^/g. The equilibrium adsorption capacity of 5% AC-MIL-101(Cr) is about 600 cm^3^/g, the maximum adsorption capacity is 866.6 cm^3^/g. The equilibrium adsorption capacity of 10% AC-MIL-101(Cr) is about 750 cm^3^/g, and the maximum adsorption capacity is 947.6 cm^3^/g. The BET specific surface area and pore volume of MIL-101(Cr) and AC-MIL-101(Cr) are calculated as shown in Table 1. The BET specific surface area of MIL-101(Cr) is 814 m^2^/g, pore volume 0.475 cm^3^/g; when the addition amount of AC is 5% and 10%, the specific surface area of 5% AC-MIL-101(Cr) and 10% AC-MIL-101(Cr) increase by 2.3 and 2.48 times, respectively. The pore volume was expanded by 2.82 times and 3.09 times, respectively. The pore size distribution curve is shown in Figure 6. MIL-101(Cr), 5% AC-MIL-101(Cr), and 10% AC-MIL-101(Cr) all have two pore structures. This finding is in good agreement with the result of the N_2_ adsorption–desorption isotherm. The addition of AC leads to defects on the crystal surface and defects in the MIL-101(Cr) unit, which helps to open the mesopores, but does not help the opening of the micropores much [21].

### 3.6. Ethanol Vapor Adsorption Isotherm

The adsorption isotherms can be used to analyze the equilibrium adsorption capacity and the interaction between the adsorbent and the refrigerant at a specific adsorption temperature. For adsorption refrigeration, the adsorption performance of the adsorbent materials should show the characteristics of rapid adsorption in the low and medium relative pressure range [22]. For the adsorption refrigeration system using ethanol vapor as the refrigerant, it is very necessary to explore the adsorption performance of the adsorbent at a relative pressure of 0.05–0.4.

**Figure 7:**
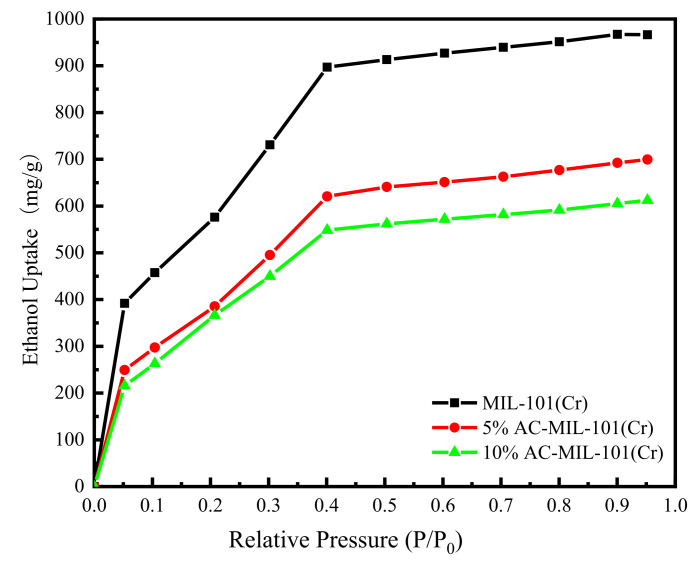
Ethanol vapor adsorption isotherms of MIL-101(Cr) and AC-MIL-101(Cr) at 303 K.

Figure 7 shows the ethanol vapor adsorption isotherm of MIL-101(Cr) and AC-MIL-101(Cr) at 303 K. It can be seen from the figure that the ethanol vapor adsorption isotherms of the three materials present “type I” adsorption isotherms [12]. The main adsorption interval of MIL-101(Cr) and AC-MIL-101(Cr) is at a relative pressure of 0–0.4. Moreover, when the relative pressure is 0–0.05, all three materials exhibit rapid adsorption ability. This phenomenon can be attributed to the CUS (coordination of unsaturated metal sites) [11,23]. When the relative pressure is 0.05 (the evaporation temperature is 283 K), the adsorption capacity of MIL-101(Cr), 5%AC-MIL-101(Cr), 10% AC-MIL-101(Cr) for ethanol vapor is about 0.4 g/g, 0.25 g/g, 0.22 g/g, respectively. When the relative pressure is 0.4 (the evaporation temperature is 288 K), the adsorption capacity of MIL-101(Cr), 5%AC-MIL-101(Cr), 10% AC-MIL-101(Cr) for ethanol vapor is about 0.9 g/g, 0.62 g/g, 0.55 g/g, respectively. At 303 K, the saturated adsorption capacity of MIL-101(Cr) for ethanol vapor is about 0.97 g/g, the saturated adsorption capacity of 5% AC-MIL-101(Cr) for ethanol vapor is about 0.7 g/g, and 10% AC-MIL-101(Cr) for ethanol vapor is about 0.61 g/g. The ethanol vapor saturated adsorption capacity of MIL-101(Cr) prepared in this study is slightly lower than the results reported in the literature [10]. It is caused by the heating rate, purification, pressure, etc., [24]. Compared with the pure sample MIL-101(Cr), the saturated adsorption capacity of 5% AC-MIL-101(Cr) is reduced by 28%, and 10% AC-MIL-101(Cr) dropped by 37%. The results show that MIL-101(Cr) and AC-MIL-101(Cr) can be better used in ice making and refrigeration conditions.

### 3.7. Adsorption Kinetics of Ethanol Vapor

**Figure 8:**
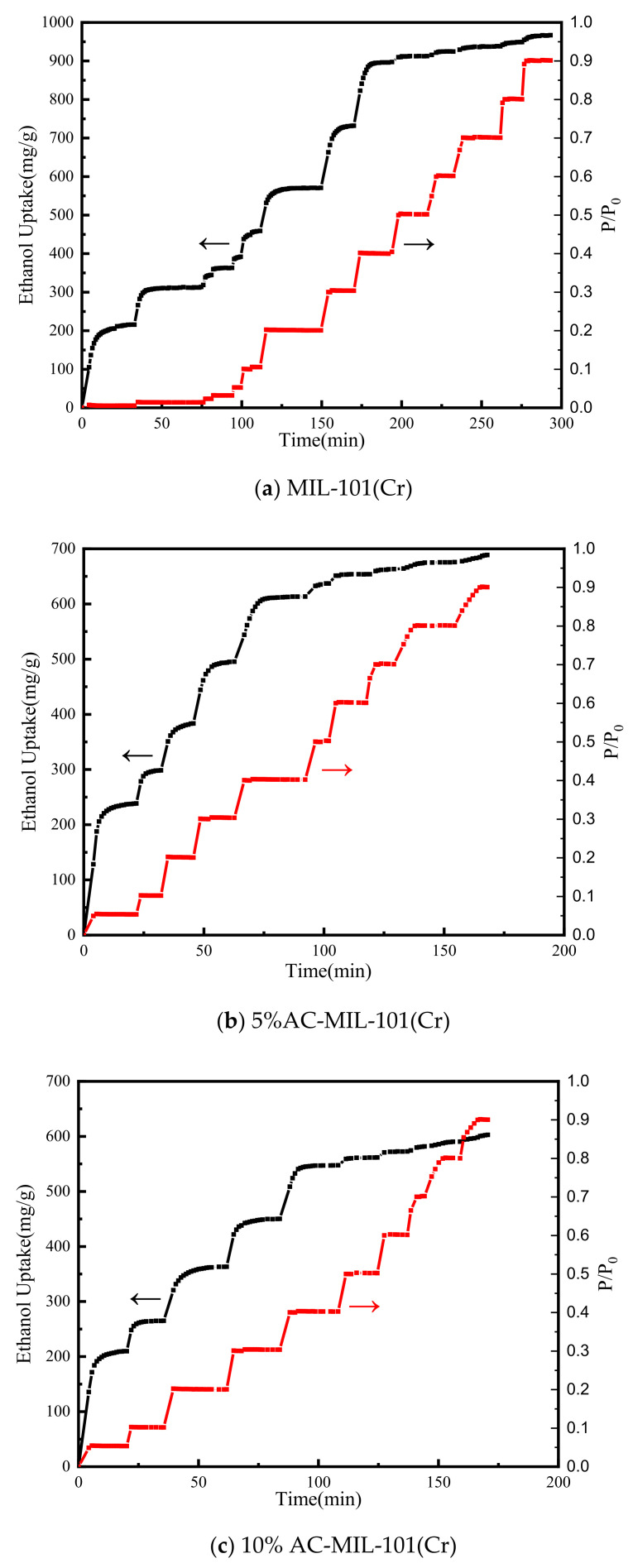
Ethanol vapor adsorption kinetic curves of adsorbent materials at 303 K: (**a**) MIL-101(Cr), (**b**) 5% AC-MIL-101(Cr), (**c**) 10% AC-MIL-101(Cr).

This work uses adsorption kinetic analysis to study the adsorption rate of adsorbent materials. In an actual adsorption refrigeration system, the speed of adsorption will directly affect the SCP of the adsorption material in the system. Therefore, it is necessary to study the adsorption kinetics of adsorbents. Figure 8 shows the ethanol vapor adsorption kinetic curves of MIL-101(Cr), 5% AC-MIL-101(Cr), and 10% AC-MIL-101(Cr) at 303 K. Observation shows that the adsorption of ethanol vapor by the three materials is mainly concentrated at a relative pressure of 0–0.4, which corresponds well to the results shown by the ethanol vapor adsorption isotherm. When the relative pressure of AC-MIL-101(Cr) is 0.05, the adsorption rate is higher. In other pressure ranges, AC-MIL-101(Cr) shows a uniform adsorption rate.

## 4. Conclusions

In this study, MIL-101(Cr), 5% AC-MIL-101(Cr) and 10% AC-MIL-101(Cr) were successfully prepared by the hydrothermal method. XRD, SEM, TGA, and N2 adsorption/desorption isotherms were performed on MIL-101(Cr) and AC-MIL-101(Cr), and the adsorption performance of the samples to ethanol vapor was also explored.

(1) The XRD results showed that the three samples were successfully prepared, and the addition of AC did not affect the skeletal crystal structure of MIL-101(Cr).
(2) SEM results showed that the three samples had similar regular octahedral structures, but the addition of AC would cause certain defects on the crystal surface of AC-MIL-101(Cr).
(3) Compared with MIL-101(Cr), the thermal stability of AC-MIL-101(Cr) improved.
(4) At 77K, N_2_ adsorption and desorption isotherm results showed that the specific surface area and pore volume of AC-MIL-101(Cr) significantly increased, and the three samples had two different cage structures. The addition of AC is conducive to the opening of the mesopores.
(5) When the relative pressure P/P0 ≤ 0.4, the three samples showed rapid adsorption of ethanol vapor. Although the ethanol vapor adsorption capacity of AC-MIL-101(Cr) decreased, AC-MIL-101(Cr) exhibited a more uniform adsorption rate. This illustrates the application prospect of AC-MIL-101(Cr) in adsorption refrigeration systems.

The exploration of AC-MIL-101(Cr) composite materials in this paper provides a reference for the future application of carbon-based/MOFs composite adsorbent/ethanol vapor working fluid in adsorption refrigeration.

## Figures and Tables

**Table 1:** Structural properties of table MIL-101(Cr) and AC-MIL-101(Cr).

Sample	S_BET_ m^2^/g	S_mi_ m^2^/g	S_me_ m^2^/g	V_total_ cm^3^/g	V_mi_ cm^3^/g	V_me_ cm^3^/g
MIL-101(Cr)	814	145	319	0.475	0.101	0.202
5%AC-MIL-101(Cr)	1863	508	686	1.340	0.245	0.776
10%AC-MIL-101(Cr)	2018	643	734	1.466	0.330	0.745

## Data Availability

Data sharing not available.

## References

[1] Clarke L., Eom J., Marten E.H., Horowitz R., Kyle P., Link R., Mignone B.K., Mundra A., Zhou Y. (2018). Effects of long-term climate change on global building energy expenditures. Energy Econ..

[2] Yan J., Yu Y., Xiao J., Li Y., Li Z. (2016). Improved Ethanol Adsorption Capacity and Coefficient of Performance for Adsorption Chillers of Cu-BTC@GO Composite Prepared by Rapid Room Temperature Synthesis. Ind. Eng. Chem. Res..

[3] De Lange M.F., Verouden K.J., Vlugt T.J., Gascon J., Kapteijn F. (2015). Adsorption-driven heat pumps: The potential of metal–organic frameworks. Chem. Rev..

[4] Frazzicaa A., Palombaa V., Dawoud B. (2016). Design, realization and testing of an adsorption refrigerator based on activated carbon/ethanol working pair. Appl. Energy.

[5] El-Sharkawy I.I., Saha B.B., Koyama S., He J., Ng K.C., Yap C. (2008). Experimental investigation on activated carbon–ethanol pair for solar powered adsorption cooling applications. Int. J. Refrig..

[6] Farha O.K., Yazaydin A.O., Eryazici I., Malliakas C.D., Hauser B.G., Kanatzidis M.G., Nguyen S.T., Snurr R.Q., Hupp J.T. (2010). De novo synthesis of a metal-organic framework material featuring ultrahigh surface area and gas storage capacities. Nat. Chem..

[7] Yoon M., Srirambalaji R., Kim K. (2012). Homochiral metal-organic frameworks for asymmetric heterogeneous catalysis. Chem. Rev..

[8] Horcajada P., Gref R., Baati T., Allan P.K., Maurin G., Couvreur P., Férey G., Morris R.E., Serre C. (2012). Metal–Organic Frameworks in Biomedicine. Chem. Rev..

[9] Betard A., Fischer R.A. (2012). Metal-organic framework thin films: From fundamentals to applications. Chem. Rev..

[10] Rezk A., Al-Dadah R., Mahmoud S., Elsayed A. (2013). Investigation of Ethanol/metal organic frameworks for low temperature adsorption cooling applications. Appl. Energy.

[11] Saha B.B., El-Sharkawy I.I., Miyazaki T., Koyama S., Henninger S.K., Herbst A., Janiak C. (2015). Ethanol adsorption onto metal organic framework: Theory and experiments. Energy.

[12] Ma L., Rui Z., Wu Q., Yang H., Yin Y., Liu Z., Cui Q., Wang H. (2016). Performance evaluation of shaped MIL-101–ethanol working pair for adsorption refrigeration. Appl. Therm. Eng..

[13] de Lange M.F., van Velzen B.L., Ottevanger C.P., Verouden K.J.F.M., Lin L.C., Vlugt T.J.H., Gascon J., Kapteijn F. (2015). Metal-Organic Frameworks in Adsorption-Driven Heat Pumps: The Potential of Alcohols as Working Fluids. Langmuir.

[14] Teo H.W.B., Chakraborty A., Kayal S. (2017). Evaluation of CH_4_ and CO_2_ adsorption on HKUST-1 and MIL-101(Cr) MOFs employing Monte Carlo simulation and comparison with experimental date. Appl. Therm. Eng..

[15] Yu Z., Deschamps J., Hamon L., Prabhakaran P.K., Pré P. (2017). Modeling hydrogen diffusion in hybrid activated carbon-MIL-101(Cr) considering temperature variations and surface loading changes. Microporous Mesoporous Mat..

[16] Yang J., Zhao Q., Li J., Dong J. (2010). Synthesis of metal–organic framework MIL-101 in TMAOH-Cr(NO_3_)_3_-H2BDC-H_2_O and its hydrogen-storage behavior. Microporous Mesoporous Mat..

[17] Gao S., Feng T., Feng C., Shang N., Wang C. (2016). Novel visible-light responsive Ag/Ag Cl@MIL-101 hybrid materials with synergistic photocatalytic activity. J. Colloid Interface Sci..

[18] Wu H., Chua Y., Krungleviciute V., Tyagi M., Chen P., Yildirim T., Zhou W. (2013). Unusual and highly tunable missing-linker defects in zirconium metaleorganic framework UiO-66 and their important effects on gas adsorption. Am. Chem. Soc..

[19] Rallapalli P.B.S., Raj M.C., Patil D.V., Prasanth K.P., Somani R.S., Bajaj H.C. (2013). Activated carbon @ MIL-101(Cr): A potential metal-organic framework composite material for hydrogen storage. Int. J. Energy Res..

[20] Yu Z., Deschamps J., Hamon L., Prabhakaran P.K., Pré P. (2017). Hydrogen adsorption and kinetics in MIL-101(Cr) and hybrid activated carbon-MIL-101(Cr) materials. Int. J. Hydrog. Energy.

[21] Sun X., Xia Q., Zhao Z., Li Y., Li Z. (2014). Synthesis and adsorption performance of MIL-101(Cr)/graphite oxide composites with high capacities of n-hexane. Chem. Eng. J..

[22] Henninger S.K., Jeremias F., Kummer H., Janiak C. (2012). MOFs for Use in Adsorption Heat Pump Processes. Eur. J. Inorg. Chem..

[23] Hong D., Hwang Y., Serre C., Férey G., Chang J. (2009). Porous chromium terephthalate MIL-101 with coordinatively unsaturated sites: Surface functionalization, encapsulation, sorption and catalysis. Adv. Funct. Mater..

[24] Férey G., Mellot-Draznieks C., Serre C., Millange F., Dutour J., Surblé S., Margiolaki I. (2005). A Chromium Terephthalate-Based Solid with Unusually Large Pore Volumes and Surface Area. Science.

